# Metalens‐Based Dual Light‐Sheet Fluorescence Microscopy

**DOI:** 10.1002/smtd.202402149

**Published:** 2025-07-22

**Authors:** Yuan Luo, Chun‐Chun Chang, Hung‐Chuan Hsu, Bo‐Wei Huang, Sunil Vyas, S.‐Ja Tseng, Cheng Hung Chu, Takuo Tanaka, Kuang‐Yuh Huang, Din Ping Tsai

**Affiliations:** ^1^ Institute of Medical Device and Imaging National Taiwan University Taipei 10051 Taiwan; ^2^ Graduate School of Advanced Technology National Taiwan University Taipei 106319 Taiwan; ^3^ YongLin Institute of Health National Taiwan University Taipei 10672 Taiwan; ^4^ Department of Biomedical Engineering National Taiwan University Taipei 10051 Taiwan; ^5^ Department of Mechanical Engineering National Taiwan University Taipei 10617 Taiwan; ^6^ Department of Pharmacology College of Medicine National Cheng Kung University Tainan 701 Taiwan; ^7^ Innovative Photon Manipulation Research Team RIKEN Center for Advanced Photonics Saitama 351‐0198 Japan; ^8^ Metamaterial Laboratory RIKEN Cluster for Pioneering Research Saitama 351‐0198 Japan; ^9^ Department of Electrical Engineering City University of Hong Kong Kowloon 999077 Hong Kong; ^10^ Centre for Biosystems Neuroscience and Nanotechnology City University of Hong Kong Kowloon 999077 Hong Kong; ^11^ The State Key Laboratory of Terahertz and Millimeter Waves City University of Hong Kong Kowloon 999077 Hong Kong

**Keywords:** dual‐sided illumination, fluorescence images, light‐sheet fluorescence microscopy, metalens, mice lung tissues

## Abstract

Light‐sheet fluorescence microscopy (LSFM) provides optically sectioned fluorescence images with excellent background rejection for rapid and volumetric imaging. However, traditional LSFM typically relies on single‐sided illumination and the stripe artifacts due to partial obstruction or scattering of the illumination beam, resulting in the formation of shadow artifacts. Uneven illumination, particularly in non‐transparent samples, results in poor contrast in certain regions of the image and reduces image uniformity. To address this problem in compact fashion, a metalens‐based dual‐sided illumination LSFM (MDI‐LSFM) is presented, which utilizes twin light‐sheets for uniform sample illumination. This is achieved by the integration of a pair of cylindrical metalenses in LSFM, forming two identical light‐sheets from opposite sides of the sample. Through a rigorous experimental setup, the system‐level structure optimization of MDI‐LSFM is successfully demonstrated to form an engineering extension by observing ex vivo images of mice lung tissues, achieving a lateral resolution of 1.7 µm with optical sectioning capability of 6.8 µm. The approach eliminates shadow artifacts and simplifies system configuration by replacing bulky optics with compact, efficient metalenses, while achieving a large field of view, high resolution, and fast imaging. These advantages enable wide‐ranging biomedical applications for in situ tissue imaging and diagnostics.

## Graphical abstract

A compact metalens‐based dual‐sided illumination light‐sheet fluorescence microscopy is proposed due to its benefits, such as eliminating shadow artifacts and enhancing image uniformity using twin light‐sheets formed by paired cylindrical metalenses. The system enables high‐resolution, large field‐of‐view tissue imaging with a simplified experimental setup, offering broad applicability for biomedical imaging and integration into commercial microscopes.

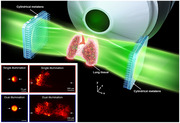

## 1 Introduction

Light‐sheet fluorescence microscopy (LSFM) has become a potential imaging system for high‐resolution and optical sectioning images of volumetric tissues.^[^ ^1^ ^]^ It provides an efficient and practical method to measure cellular and developmental biological processes.^[^ ^2^, ^3^ ^]^ LSFM illuminates a thin sheet of light to excite fluorescently labeled samples from the side, and its emitted fluorescence images are acquired orthogonally to the illumination axis.^[^ ^4^ ^]^ Compared to confocal microscopy,^[^ ^5^ ^]^ the orthogonal configuration between illumination and detection offers multiple advantages, including minimal phototoxicity, fine optical sectioning, and high‐speed image operation.^[^ ^6^, ^7^ ^]^ However, the unique geometric constraints produce many challenges from cumbersome optical components for detection and illumination. The sample holder is positioned and integrated into LSFM within a limited physical space for high‐resolution imaging performance.^[^ ^8^ ^]^

Standard LSFM, utilizing single‐sided illumination, suffers from strong shadow artifacts that are commonly associated with stripe artifacts caused by partial obstruction or scattering of the illumination beam,^[^ ^9^, ^10^, ^11^ ^]^ which severely degrade image uniformity and limit the system field of view (FOV).^[^ ^12^ ^]^ Various illumination schemes have been proposed to increase system FOV and reduce the thickness of light‐sheets to observe fine features of tissues.^[^ ^13^, ^14^, ^15^, ^16^, ^17^, ^18^ ^]^ Some recent examples are based on paired objective lenses in multiple excitation directions,^[^ ^19^, ^20^, ^21^ ^]^ and wavefront shaping to extend system FOV in fast volumetric imaging. The former approach requires many cumbersome optical components to enlarge FOV,^[^ ^22^, ^23^, ^24^, ^25^ ^]^ while the latter uses diffractive optics or passive spatial light modulators to manipulate the light field. Unfortunately, all these variants require complex designs and bulky optical elements.

Metasurfaces, a class of engineered nanostructures, provide multiple advantages over refractive optics in terms of flat shape, lightweight, and ultracompact size.^[^ ^26^, ^27^, ^28^, ^29^, ^30^ ^]^ Their unique properties arise from subwavelength features that manipulate light fields with high precision. Various fundamental optical elements, including lenses, wave plates, polarizers, and holograms, have been realized using metasurfaces, often with additional functionalities like broadband operation, achromatic behavior, and structured illumination.^[^ ^31^, ^32^, ^33^, ^34^, ^35^ ^]^ Over the past decade, advancements in fundamental understanding and fabrication techniques have driven the development of efficient metalenses across the visible and infrared spectrum.^[^ ^36^, ^37^, ^38^, ^39^, ^40^ ^]^ The design freedom and material selection make metalens attractive for a broad range of applications.^[^ ^41^, ^42^, ^43^, ^44^, ^45^ ^]^ Recently, there have been a few reports on metalens‐based microscopy.^[^ ^46^, ^47^, ^48^, ^49^, ^50^ ^]^ However, fluorescence optical sectioning microscopy with high resolution, large FOV, and rapid acquisition for volumetric tissue imaging has not been explored so far.

In this article, a metalens‐based dual‐sided illumination LSFM (MDI‐LSFM) system is experimentally demonstrated for imaging ex vivo mice lung tissues. In our approach, paired cylindrical metalenses are implemented with a sample holder in LSFM to form an engineering extension of the traditional approach, and uniformly generate twin light‐sheets for simultaneously exciting fluorescently labeled tissues from opposite sides. The cylindrical metalenses are designed with a measured transmission efficiency of 44.3%, which provides different modular options depending on the applications. The MDI‐LSFM overcomes the above‐mentioned shadow artifacts and complicated system configuration while providing extended system FOV and optically sectioned fluorescence images with excellent background rejection for rapid volumetric imaging. We experimentally evaluate the image uniformity and system characteristics of the MDI‐LSFM, achieving a sub‐cellular resolution of 1.1 µm with optical sectioning capability of 6.8 µm. In addition, image quality is significantly enhanced by the Richardson‐Lucy (RL) deconvolution method.^[^ ^51^ ^]^ To our knowledge, this is the first report on applying paired metalenses in dual‐sided illumination LSFM. Our approach advances imaging fields of metasurfaces in rapid, large FOV, and high‐resolution fluorescence microscopy, which has great potential for volumetric tissue imaging under the miniaturization of such optical systems.

## 2 Results

### 2.1 Design of Paired Cylindrical Metalenses

**Figure 1:**
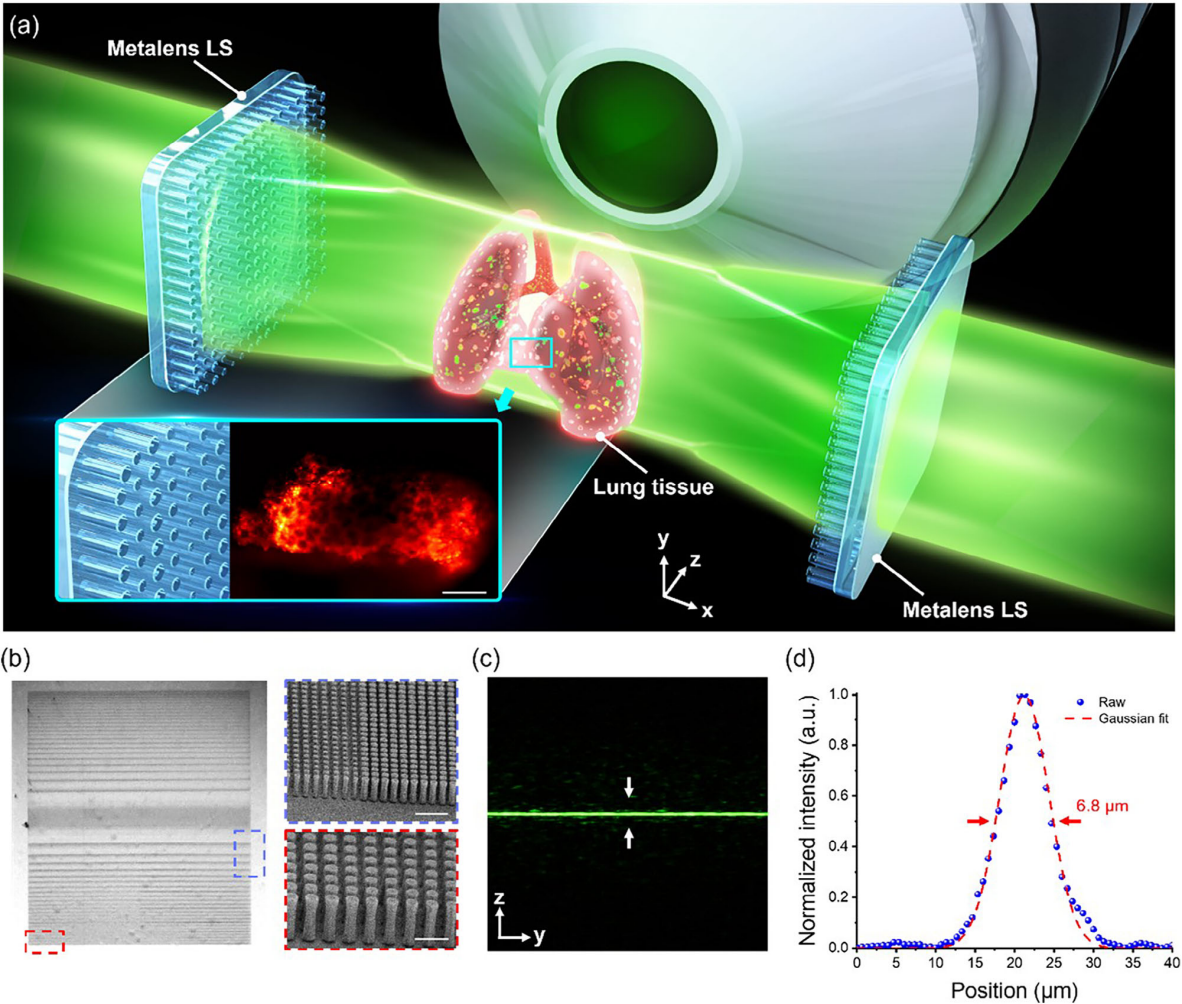
The schematic diagram of the MDI‐LSFM. a) The paired cylindrical metalenses are used for dual light‐sheet illumination. The inset in the diagram illustrates the enlarged regions of nanopillars and the measured lung tissue slice with a scale bar of 200 µm. b) An image of the fabricated cylindrical metalens using a wide‐field microscope. Zoomed‐in corresponding subset SEM images to the blue dashed‐box region (with a scale bar of 1 µm) and red dashed‐box region (with a scale bar of 0.5 µm). c) Experimental measurement of the intensity profile from the metalens at the focal plane. d) The intensity profile and Gaussian fitting along the white arrows in (c).

The schematic diagram of the proposed MDI‐LSFM system is illustrated in **Figure** **1**a, and the enlarged inset illustrates the measurement of sectioned mouse lung tissue to reveal fine structural details. The parameters of the phase mask are selected through simulation for the practical implementation of the metalens in MDI‐LSFM. More details about the simulation results can be found in Section S1 (Supporting Information). The GaN‐based metalenses are chosen due to their high transparency and excellent performance in modulating the phase.^[^ ^29^, ^52^ ^]^ A 450 µm‐thick square‐shaped Al_2_O_3_ substrate and the dielectric GaN circular nano‐antennas with a height of 850 nm are utilized to provide complete 2*π* phase control. The commercial software CST Studio Suite is utilized to simulate the various parameters, such as phase delay and transmission efficiency of GaN circular nano‐antennas. The circular nano‐antennas are designed with diameters ranging from 100 to 185 nm and a pitch of 280 nm. Details of the CST simulation are presented in Section S2 (Supporting Information). The circular symmetry from the unit cell also provides polarization insensitivity, which can be used in more diverse applications. More results under the incident light of x and y polarization and illumination with different wavelengths are shown in Sections S3 and S4 (Supporting Information). Figure 1b shows an image of the metalens taken by a wide‐field optical microscope, and two subset figures show corresponding zoomed‐in images of nanorods using a scanning electron microscope (SEM) at different locations.

Under incident light along the *z*‐direction, the phase distribution *φ(x,y)* of the cylindrical metalenses for MDI‐LSFM is designed under the thin lens approximation, and can be written as

(1) $$\varphi \left(x,\;y\right)=\frac{2\pi }{\lambda }\left(\sqrt{x^{2}+f^{2}}-f\right)$$

where *f* represents the focal length of the metalens, and *λ* is the wavelength of light utilized for illumination. The designed parameters for paired identical metalenses in MDI‐LSFM include aperture dimensions of *D* = 1 mm, focal length of *f* = 10 mm, central operation wavelength of *λ* = 532 nm, and numerical aperture of *NA* = 0.05. In contrast to conventional optical elements that rely on refraction to control light phase for convergence and divergence, the metalenses utilize different resonant modes of discretely distributed nano‐antennas to adjust the required phase shift. The intensity distribution at the focal plane from the cylindrical metalens is experimentally obtained, as demonstrated in Figure 1c. On the other hand, to produce a light‐sheet, a configuration of a conventional objective lens and a cylindrical lens needs 115 mm long, and even a standalone cylindrical lens still occupies 15 mm, as shown in **Table** **1**. By exploiting the phase‐modulation capability of nanopillars, our cylindrical metalens delivers the same functionality with a thickness of merely 800 nm, which highlights the modular and engineering advantages of meta‐optics technology. In our measurement of both cylindrical metalenses, we can also achieve the FOV of 152 ± 1 µm. More details about the experimental configuration for measuring the beam profile are shown in Sections S5 and S6 (Supporting Information). The intensity profile in the focal plane also follows the theoretically predicted intensity distribution. A Gaussian‐like intensity distribution is shown in Figure 1d, which demonstrates that the light‐sheet generated by the cylindrical metalens is reasonably thin with a full width at half maximum (FWHM) of 6.8 µm.

**Table 1:** Comparison between conventional lenses and metalens.

	Objective lens + Cylindrical lens^a)^	Cylindrical lens	Cylindrical metalens
Size (x × y × z)	32.2 mm × 32.2 mm × 115 mm	25.4 mm × 25.4 mm × 15 mm	1 mm × 1 mm × 800 nm
Focal length	40 mm	75 mm	10 mm
FOV	32 µm	40 µm	152 µm
Diameter	25.4 mm	25.4 mm	1 mm
Required component	2	1	1

^a)^ The parameters of the last optical component.

### 2.2 The System Verification of MDI‐LSFM

**Figure 2:**
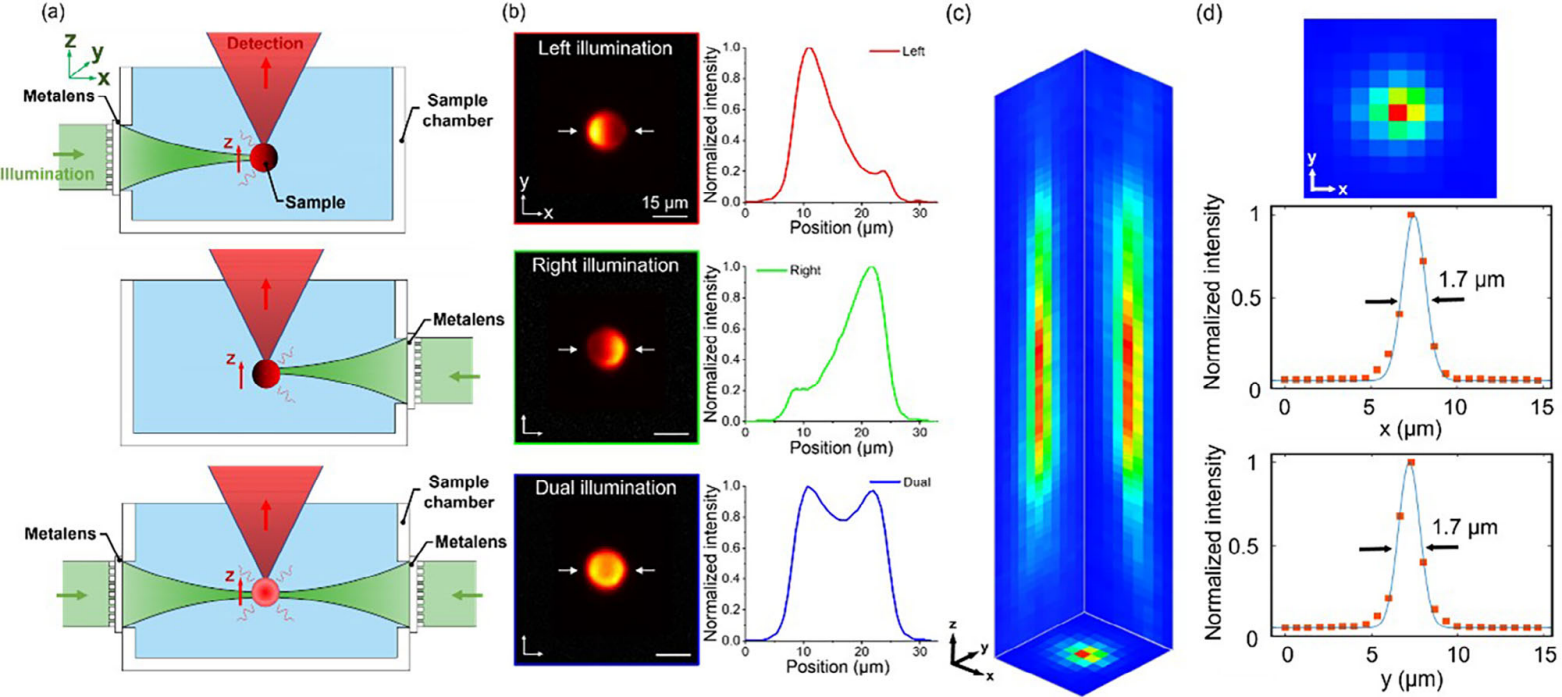
Fluorescent imaging of a microsphere using MDI‐LSFM. a) System configurations of the MDI‐LSFM under single‐sided from left and right, as well as dual‐sided excitation. b) Imaging performance and intensity cross‐section profiles to observe the 15‐µm fluorescent microsphere under single‐sided illumination and dual‐sided illumination. c) The measured PSF using a 1‐µm fluorescent microsphere. d) The top part shows intensity distribution along the *x‐y* plane of the PSF. The middle and bottom parts are the line profile along the *x‐*axis and *y‐*axis of the PSF in the *x‐y* plane.

To experimentally compare the shadow artifacts in conventional LSFM and demonstrate how our proposed MDI‐LSFM addresses this issue, a single fluorescent bead has been imaged under single‐sided and double‐sided illumination, as shown in **Figure** **2**a. The figure depicts metalens for illumination, a sample chamber, and detection, in which the mitigation of shadowing effects achieved by MDI‐LSFM is directly observed and analyzed. The measured results under the single‐sided and dual‐sided illumination are illustrated in Figure 2b by imaging a fluorescent microsphere with a diameter of 15 µm at the emission wavelength of 605 nm. The fluorescent microspheres are embedded in a transparent, low‐concentration (≈0.5%) agar gel, which provides refractive index matching and a suitable environment for live samples. To demonstrate the performance of the MDI‐LSFM system, the point spread function (PSF) is also measured by imaging a red fluorescent bead with a diameter of 1.0 µm, which is positioned in agar within a water container, as shown in Figure 2c. The use of beads with this diameter is chosen due to the NA of the detection arm being ≈0.3. The theoretical lateral resolution is calculated to be 1.23 µm, and the axial resolution depends on the light‐sheet thickness, as mentioned previously. The piezo stage is used to move along the *z*‐axis with a step size of 0.5 µm, enabling the acquisition of fluorescence images of the samples at varying depths. In Figure 2d, the orange data points represent experimental measurements of the PSF along the *x*‐axis and *y*‐axis, while the blue line represents an approximation curve. The directions of both the x‐axis and *y*‐axis exhibit similar point spread functions, with FWHM of ≈1.7 µm. The fluorescence images of microspheres demonstrate the effectiveness of dual‐sided illumination in achieving uniform illumination. Furthermore, the MDI‐LSFM can be extended beyond dual‐sided illumination to a multi‐sided illumination system by incorporating additional lenses.

**Figure 3:**
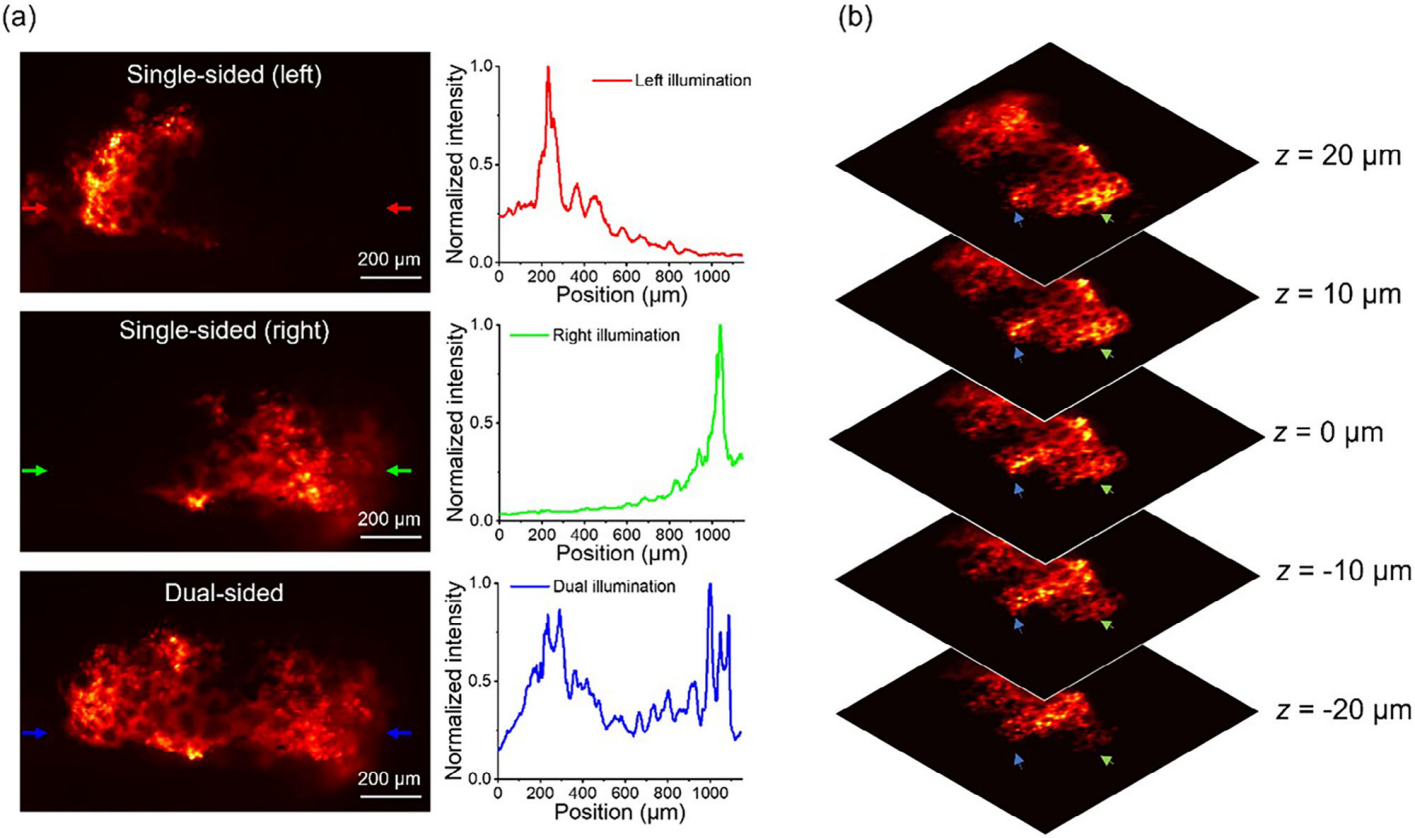
Ex vivo images of fluorescent mice lung tissue with single‐sided and dual‐sided excitation. a) The figures on the left show optically sectioned images of the mice lungs using single‐sided and dual‐sided illumination. The figures on the right show the corresponding cross‐section profiles along the arrows. b) Stack of five MDI‐LSFM images of the lung tissue at different depths.

To validate the imaging capability of MDI‐LSFM for biological samples, ex vivo mice lung tissues doped with the Propidium Iodide and PbS core‐type quantum dots are used as shown in **Figure** **3**. For ex vivo imaging, lung tissues are harvested immediately after the mice are sacrificed.^[^ ^53^, ^54^ ^]^ The lungs are kept intact and then cut into 3 to 5 mm‐thick tissue slices. All animal experiments are conducted according to the guidelines and protocols approved by the Health and Safety and Environment Office of the National Taiwan University. Figure 3a shows the resultant images of ex vivo lung tissues, measured by both single‐sided and dual‐sided excitation. In comparison, images with single‐sided illumination are insufficient to acquire the entire image of the fluorescently labeled lung sample, while images with dual‐sided illumination are highly effective in obtaining rapid optically sectioned images with larger FOV and faster acquisition. The intensity profiles along the arrows are highlighted in different colors on the left in Figure 3a, which also shows the comparison between single‐sided and dual‐sided illumination, as demonstrated on the right of Figure 3a. Figure 3b shows resultant MDI‐LSFM stack images of fluorescent lung tissue from depths of ‐20 to 20 µm. The stack images at different depths demonstrate fine optical sectioning ability and minimal shadow effect within volumetric lung tissues obtained from the MDI‐LSFM system.

Due to the inhomogeneity of the refractive index and variation in the absorption and scattering across the biological sample, fluorescence images contain some noise, which limits the image quality and contrast.^[^ ^55^ ^]^ The unwanted background noise can be removed using deconvolution methods. In this work, the Richardson–Lucy (RL) deconvolution method is used to eliminate the background noise.^[^ ^51^, ^56^ ^]^ This method is based on iterative optimization, incorporating the measured PSF and maximum likelihood estimation. For the experimentally acquired image *I_0_* and PSF *h*, the image obtained through the n‐th iteration can be represented as

(2) $$I_{n+1}=I_{n}\cdot \left(\frac{I_{0}}{I_{n}⊗h}⊗h^{*}\right)$$

where ⊗ denotes the convolution operation, and ^*^ represents the image flipping. The initial value of the image is taken as *I_1_* = *I_0_*. The PSF for the deconvolution is experimentally acquired from the slices of a 1 µm microsphere by scanning the fluorescent microsphere‐embedded sample with a linear stage along the *z*‐direction. Final images are acquired by deconvolving the images with the experimentally measured PSF.

**Figure 4:**
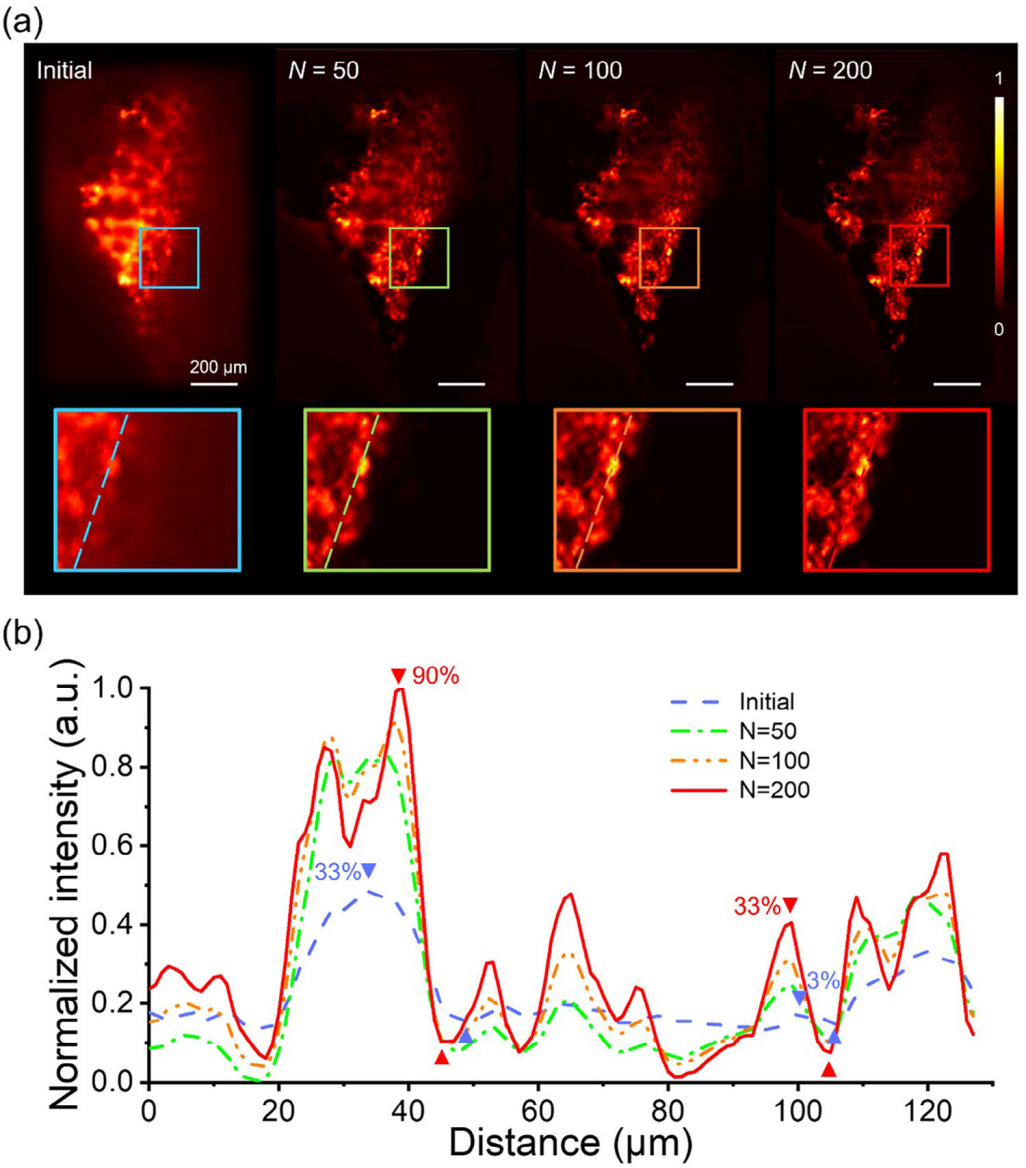
High‐resolution images of mice lung tissues using the RL deconvolution method. a) Image results of the lung tissue for iterative numbers of 0 (Initial), 50, 100, and 200. The inset figures show zoomed‐in images of the blocked region. b) Intensity cross‐section profiles are compared for iterative numbers of 0, 50, 100, and 200 along the dashed lines in (a).

The RL deconvolution method utilizes the measured PSF to iteratively process blurred and noisy images. The DeconolutionLab2 package, developed by Sage et al.,^[^ ^57^, ^58^ ^]^ is utilized to improve the image quality. **Figure** **4**a illustrates the initial and deconvolved images of mice lung tissues with iteration counts of *N* = 50, 100, and 200. After deconvolution, the zoomed‐in images corresponding to the top left part show more detailed structures due to the removal of the out‐of‐focus noise.

By appropriately selecting the iteration number and calculating the contrast using the equation:

(3) $$Contrast=\left(Max-Min\right)/\left(Max+Min\right)$$

where *Max* represents the maximum and *Min* denotes the minimum value within the region of interest (ROI), image contrast can be improved from 33% to 90% at the distance range of 25–50 µm, and from 3% to 33% within the distance range of 95–110 µm. This adjustment leads to a significant improvement in image quality, as shown in Figure 4b. The arrow regions on the relative intensity profiles depict that more features become observable with deconvolution, enhancing imaging contrast. However, excessive iterations may lead to image distortion and the generation of artifacts. In combination with the deconvolution method, MDI‐LSFM provides two advantages: first, it provides better uniform illumination of regions of interest inside the samples, and second, it enhances contrast using experimentally measured PSFs. Zoomed‐in high‐contrast 3D images of mice lung tissue, visualized using MDI‐LSFM and the deconvolution method, reveal finer features that are not visible due to shadowing effects with conventional methods.

**Figure 5:**
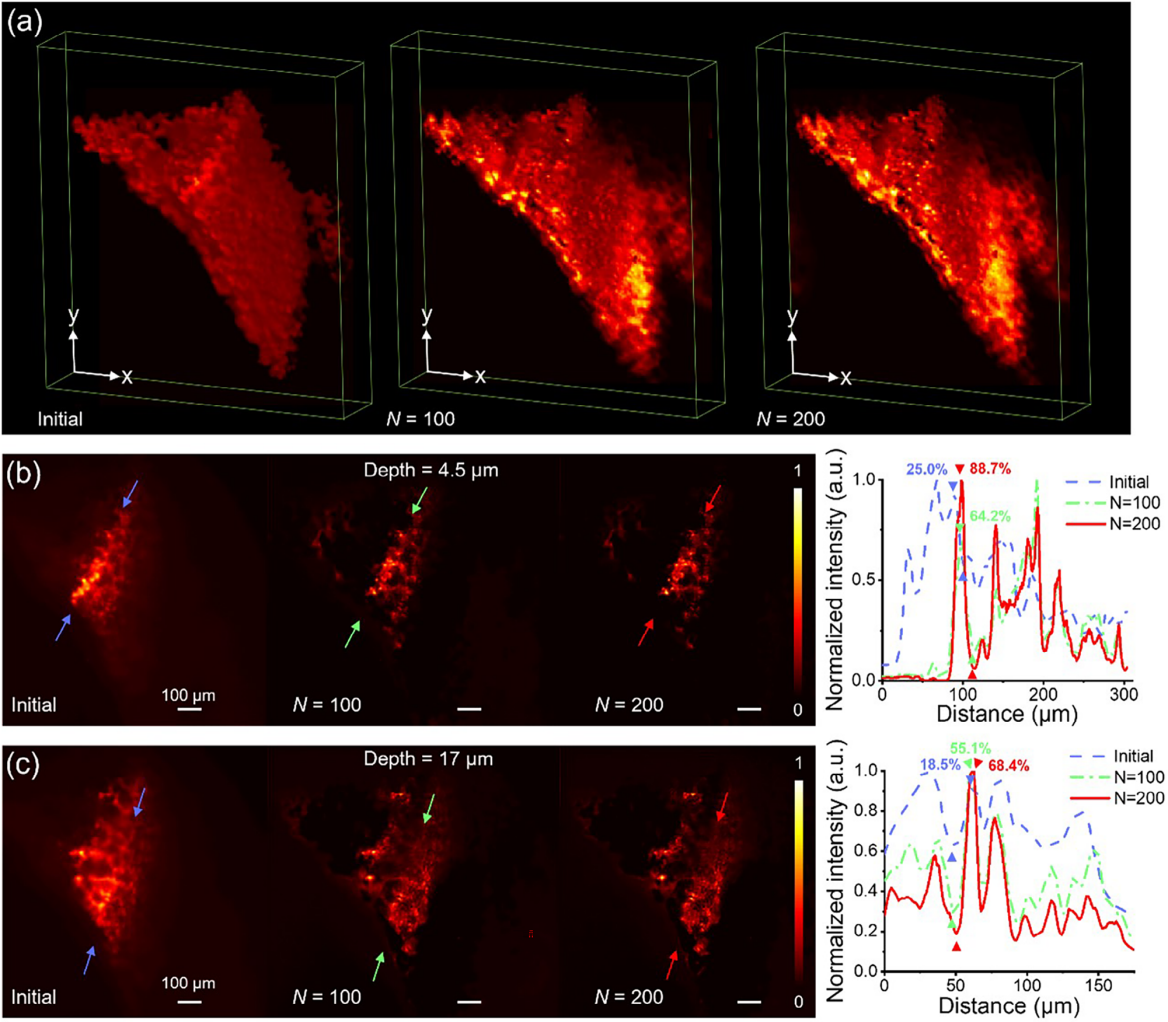
Volumetric image stacks of mice lung tissues at different depths. a) The initial and restored volumes of mice lung tissues using deconvolution with iterative numbers of 100 and 200. b) The images of mice lung tissues at a depth of 4.5 µm. The right figure compares the initial image with those at iterative numbers of 100 and 200. c) The images of the lung tissues at a depth of 17 µm. The right figure compares the initial with those at iterative numbers of 100 and 200.

The other features, otherwise shadowed due to high absorption and scattering from the sample, can be recovered by utilizing the RL deconvolution method, and a large FOV with high image contrast can be obtained. **Figure** **5**a shows significant contrast improvement in the measured volume after applying the RL deconvolution method. The enhanced image quality of restored images at a depth of 4.5 µm is shown in Figure 5b, where the contrast is improved from 25.0% to 64.2% and 88.7% with the iterative number increasing from 0 to 100 and 200. Figure 5c shows the improved results at a depth of 17 µm, where the image contrast is also enhanced from 18.5% to 55.1% and 68.4% with the iterative number from 0 to 100 and 200, revealing finer features of the mice lung tissues. By combining the advantages of paired metalenses for dual‐sided excitation, our MDI‐LSFM system provides image resolution at the cellular level for large FOV and uniform illumination. The lateral resolution of the MDI‐LSFM system is limited by the NA of the lens in the detection arm. In our case, the water‐immersion objective (10 × Olympus UMPLFLN, Olympus) with an average light wavelength of *λ_W_* = 500 nm and a working distance of 3.5 mm (NA≈0.3) is used, resulting in a measured lateral resolution in our detection arm of 1.7 µm, which is closely related to the theoretical resolution of ≈1.23 µm. Therefore, resultant images obtained using the MDI‐LSFM show solid evidence to perform high‐resolution, large FOV, and rapid acquisition of optically sectioned images.

## 3 Conclusion

In summary, our approach successfully acquires high‐resolution optically sectioned images of volumetric tissue samples, enabling large FOV and rapid acquisition for in situ tissue imaging. The system‐level structural optimization of MDI‐LSFM is experimentally compared to conventional single‐sided excitation, and the resultant images of standard fluorescent microspheres and lung tissues demonstrate solid advantages. The MDI‐LSFM overcomes shadow artifacts and simplifies the system configuration with ultra‐compact paired metalenses. Metalens‐based dual‐sided excitation, incorporating the design of the sample holder in LSFM, can be fabricated as an add‐on module for any commercial microscope. Metalenses for shaping illumination are both miniature‐sized and design‐flexible, making them ideal for other microscope modalities where the instrument's size and shape are crucial. The illumination scheme presented here provides a simple and robust method that is easy to use in any commercial or homemade microscope. Although paired cylindrical metalenses are designed as an example, different beam shapes with more functionality can be easily incorporated. The broadband operation capability of metalenses may further allow the proposed system to observe deep tissue imaging for near‐infrared (NIR) fluorescent imaging. We believe the intrinsic design flexibility, broadband operation, and compact size make metalens ideal candidates for advanced microscopic systems to provide a variety of biomedical applications.

## 4 Experimental Section

### Fabrication Process for the Cylindrical Metalens

The fabrication process for the desired metalens with the sequential deposition of an 800 nm GaN via metalorganic chemical vapor deposition (MOCVD) and a 400 nm SiO_2_ via plasma‐enhanced chemical vapor deposition (PECVD) onto a sapphire substrate. Subsequently, an ≈150 nm thick diluted positive photoresist (ZEP‐520A:ZEPA = 1:1) was spread over the SiO_2_ layer at room temperature by utilizing the spin coater. After that, the designed phase pattern of the cylindrical lens was drawn by using electron beam lithography. After exposure, the desired phase mask was obtained utilizing a developer solution (ZED‐N50). The final pattern was formed on the SiO_2_ layer by reactive ion etching (RIE), with chromium (Cr) of ≈35 nm thickness used as an additional etching hard mask. BCl_3_/Cl_2_ with ICP‐RIE mixtures was used to etch the desired pattern of GaN circular nanopillars. The GaN metalens with the phase distribution of the cylindrical lens was ultimately achieved after the residual SiO_2_ hard mask was removed.

### Experimental Setup

A diode‐pumped solid‐state laser (Genesis MX532‐1000 SLM, Coherent) with an operation wavelength at 532 nm provides the illumination beam for excitation. The input light beam was guided through optical fibers to form dual‐sided illumination through the paired cylindrical metalenses attached to the sample chamber's opposite sides. Ex vivo lung tissue samples were immersed in the liquid within the sample holder (215 × 135 × 100 mm^3^). A depth scanning device consisting of the piezo scanning stage (models Q‐545.140 and PI E‐871, Physik Instrumente) was utilized to move along the *z*‐axis with a step size of 0.5 µm. The detection arm of the microscope incorporates an Olympus BX51 microscope coupled with the water immersion objectives of various magnifications. At the illumination end, light was guided through optical fibers, split using a beam splitter, and reflected by the mirrors to form a dual beam for the metalens. The entire illumination setup was adjustable in terms of position through a *z*‐axis elevation platform and an x‐y linear stage. The illumination and sample setup size can be limited to 215 × 135 × 100 mm^3^. The microscope detector magnifies and images the emitted fluorescent light onto the camera (Hamamatsu ORCA‐ER, 12 bit, 1344 × 1024 pixels). A home‐built LabVIEW automatic control platform captures image signals while synchronously controlling the depth scanning device and camera. The image processing and 3D reconstruction were conducted using ImageJ software.^[^ ^57^ ^]^ The microscope on the detection arm was fixed to a base, while the illumination generated by the light‐sheet can be adjusted at the focal plane of the detection objective. The sample undergoes depth scanning along the *z*‐axis for 3D imaging. It was essential to prevent the metal nanostructures from coming into the water to avoid a change in the predetermined optical path length. The depth scanning actuator moves the sample in the *z*‐direction through the sample holder, with an opening above the sample holder allowing the detection objective to capture fluorescent signals. After passing through the metalens, the two illumination beams form the dual‐sided illumination light‐sheet. More details about the experimental setup for the proposed MDI‐LSFM are shown in Section S7 (Supporting Information).

### Sample Preparation

All animal experiments were conducted in accordance with the guidelines specified in the “Guide for the Care and Use of Laboratory Animals” and received prior approval from the Use Committee and Institutional Animal Care from the College of Medicine at National Taiwan University. Six‐ to seven‐week‐old Male BALB/c mice were procured from the National Laboratory Animal Center in Taiwan for this study. Lung tissues were collected and analyzed, with standard procedures applied to formalin‐fixed paraffin‐embedded (FFPE) samples. Sections of lung tissue were stained using oleic acid‐coated PbS Quantum Dots (10 mg mL^−1^ in toluene) and 10 mg kg^−1^ propidium iodide (excitation/emission: 493/636 nm).

## Conflict of Interest

The authors declare no conflict of interest.

## Supporting information



Supporting Information

## Data Availability

The data that support the findings of this study are available in the supplementary material of this article.
