# Supplementary material for: Metalens‐Based Dual Light‐Sheet Fluorescence Microscopy
Source: Small Methods. 2025 Jul 22;9(9):e02149. doi: 10.1002/smtd.202402149 (PMC12464795; doi:10.1002/smtd.202402149)
Supplement: Supplementary file 1 — Supporting Information [file SMTD-9-e02149-s001.docx]

**Metalens-based dual light-sheet fluorescence microscopy**

Supporting Information

Metasurface-based dual light-sheet fluorescence microscopy

Yuan Luo*, Chun-Chun Chang, Hung-Chuan Hsu, Bo-Wei Huang, Sunil Vyas, S.-Ja Tseng, Cheng Hung Chu, Takuo Tanaka, Kuang-Yuh Huang*, Din Ping Tsai*

**S1. Simulated results of cylindrical metalens using angular spectrum method**

The angular spectrum method (ASM) is a technique used for simulating light field propagation based on the principles of Fourier optics. It is primarily utilized to model the propagation of light waves.^[1]^ This technique performs a Fourier transform on the light field at different propagation depths, decomposing it into different plane wave components. It is essential to consider the phase changes of each component during propagation, and each component must be multiplied by the spatial frequency transfer function (SFTF). Finally, an inverse Fourier transform is conducted to obtain the distribution of light field intensity on a specific plane. Equations (1) and (2) represent the mathematical expressions for ASM and SFTF, respectively.

$U\left( z,y \right)=\mathcal{F}^{-1}\left\{ \mathcal{F}\left\{ U\left( \xi, \eta\right) \right\}\mathcal{\times H}\left( f_{z},f_{y};x \right) \right\}$, (1)

$\mathcal{H}\left( f_{z},f_{y};x \right)=\exp[-j2\pi x\sqrt{1/\lambda^{2}-f_{z}^{2}-f_{y}^{2}}]$, (2)

The ASM is used to simulate the far-field results of cylindrical metalens and the propagated beam profile of the cylindrical metalens along the x-z plane, as shown in Fig. S1(a). Figures S1(b) and S1(c) display the beam propagation and intensity distribution of light along the y-z plane of the focal plane. The results are consistent with the design parameters with the wavelength λ of 532 nm, dimensions D of 1 mm × 1 mm, focal length f of 10 mm, and numerical aperture NA of 0.05.


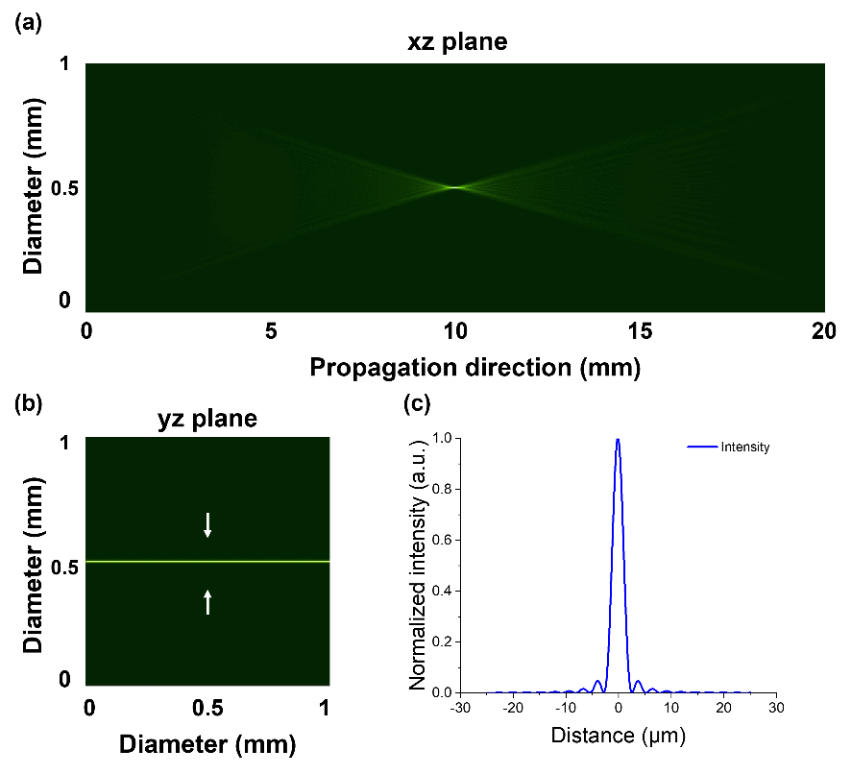


**Figure S1. The simulated beam propagation of the cylindrical metalens using the angular spectrum method has the same specifications as the experiment. (a)** The simulated beam propagation along the x-z plane. **(b)** The simulated beam propagation at the y-z plane of the focal plane. **(c)** The intensity profile along the white arrows is shown in **(b)**.

**S2. The simulated results for the metalens design and wavefront of the cylindrical lens**

By adjusting the diameters of nanopillars, the designed metalens can achieve a wide 2π phase modulation range (shown by the blue data points in Fig. S2(a)) with excellent transmission efficiency (>80%, as shown by the red data points in Fig. S2(a)). Crucially, under ambient light, the nanopillars used in this work are insensitive to polarization states and have circular symmetry. The polarization-independent property of the nanopillars eliminates the need for additional polarization elements in the cylindrical metalens. The metalens is composed of more than ten million nanopillars of varying diameters, which are carefully placed along the substrate surface to match the intended phase profile. After obtaining the transmission efficiency and 2π phase range, we simulated the phase mask of the cylindrical wavefront, as shown in Fig. 2(b).


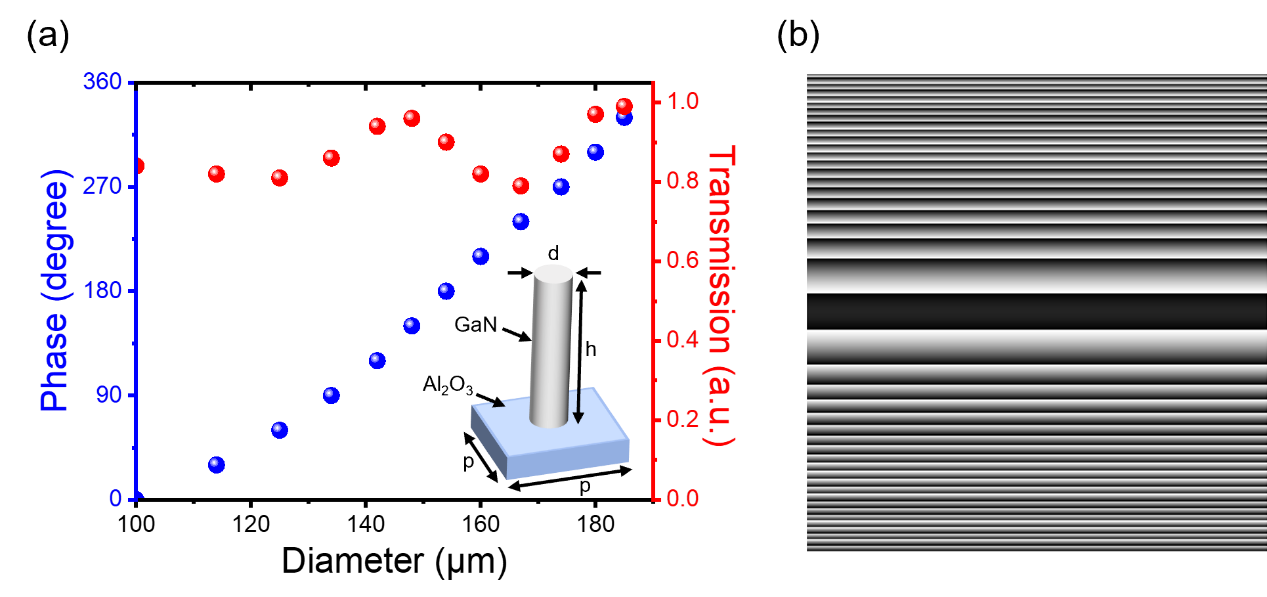


**Figure S2. The simulated 2π phase range of nanopillars and phase distribution of cylindrical metalens. (a)** Simulated results for nanopillars show varying transmission efficiency (denoted by blue points) and phase modulation (denoted by red points) across different diameters. Insert: The geometric parameters of the nanopillars, with a height h of 850 nm, a period p of 280 nm, and a diameter d ranging from 100 to 185 nm. **(b)** The phase profile of the cylindrical metalens designed to generate a thin sheet of light.

**S3. The simulated results for the cylindrical metalens under x and y polarization light**

To simulate the intensity distribution of the cylindrical metalens along the propagation direction, we utilize the simulation software CST. The parameters include a pitch of 280 μm, a height of 850 nm, and diameters ranging from 100 to 185 nm. The refractive index of the Al_2_O_3_ substrate is set to 1.77, and the complex relative permittivity of GaN is calculated using the reference.^[2-4]^ The unit cell model is configured with periodic boundary conditions in the x-y plane and open boundary conditions along the z direction. The computational simulations are performed on a computer equipped with an Intel Xeon W5-2465X processor, which has a 33.75 MB cache and a base clock speed of 3.1 GHz, capable of reaching 4.7 GHz with turbo boost, as shown in Fig. S3. Due to memory limitations, we choose a smaller overall scale while maintaining the same NA in the simulation results.^[5]^ This approach not only provides similar results but also saves unnecessary memory usage.


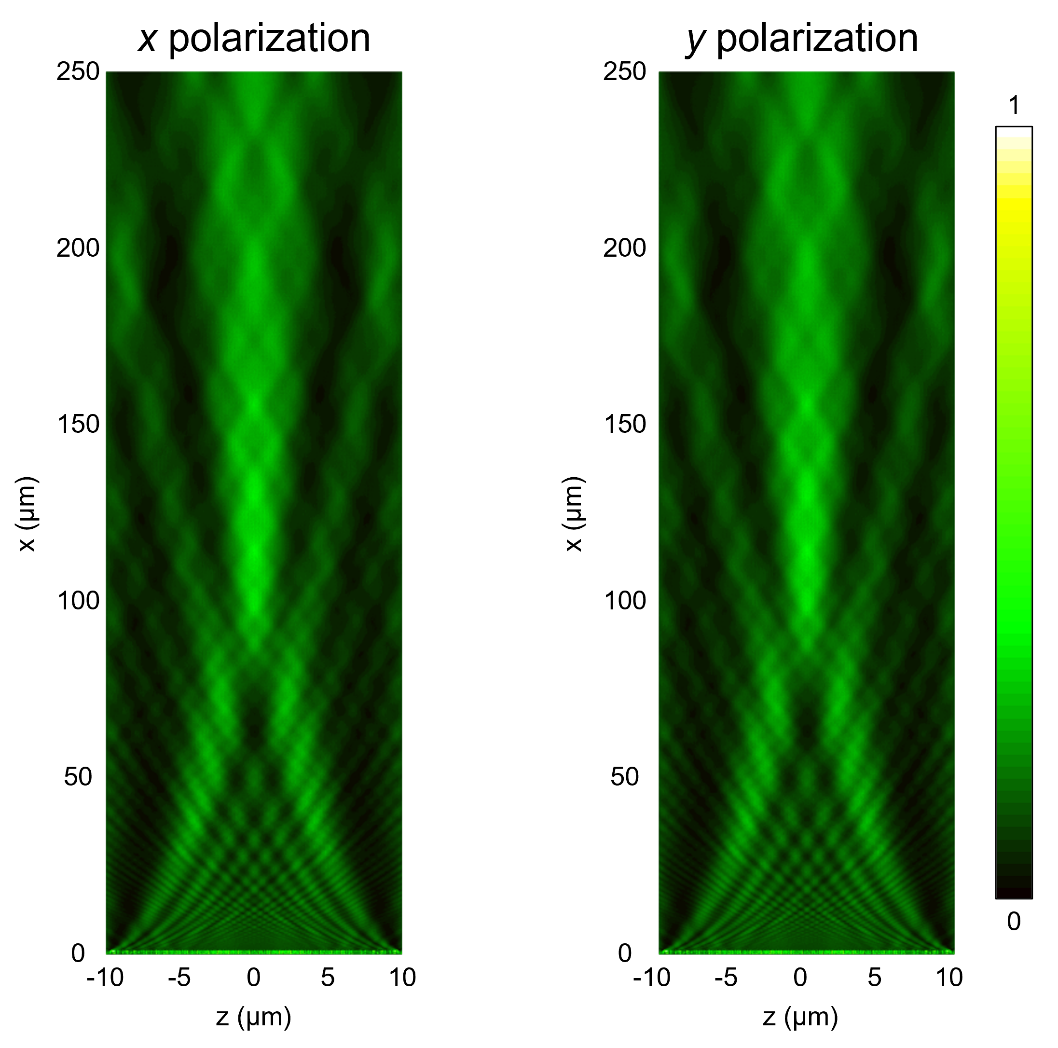


**Figure S3. The simulated intensity distribution of the cylindrical metalens under incident light of x and y polarization. (a)** Simulated results for nanopillars show varying transmission efficiency (denoted by blue points) and phase modulation (denoted by red points) across different diameters. Insert: The geometric parameters of the nanopillars, with a height h of 850 nm, a period p of 280 nm, and a diameter d ranging from 100 to 185 nm. **(b)** The phase profile of the cylindrical metalens designed to generate a thin sheet of light.

**S4. Simulated results for the cylindrical metalens under different wavelengths**

In addition to the intensity distribution of the cylindrical metalens at the operating wavelength of 532 nm, we also simulate the intensity distribution at the wavelengths of 491 nm and 632 nm. The results show the differences when compared to the simulated propagation distribution of the selected thin light-sheet at 532 nm, as shown in Fig. S4. The phenomenon of phase mismatching is observed at wavelengths different from the specifically designed wavelength, contributing to a decrease in transmission efficiency and an increase in light scattering, resulting in augmented optical noise.


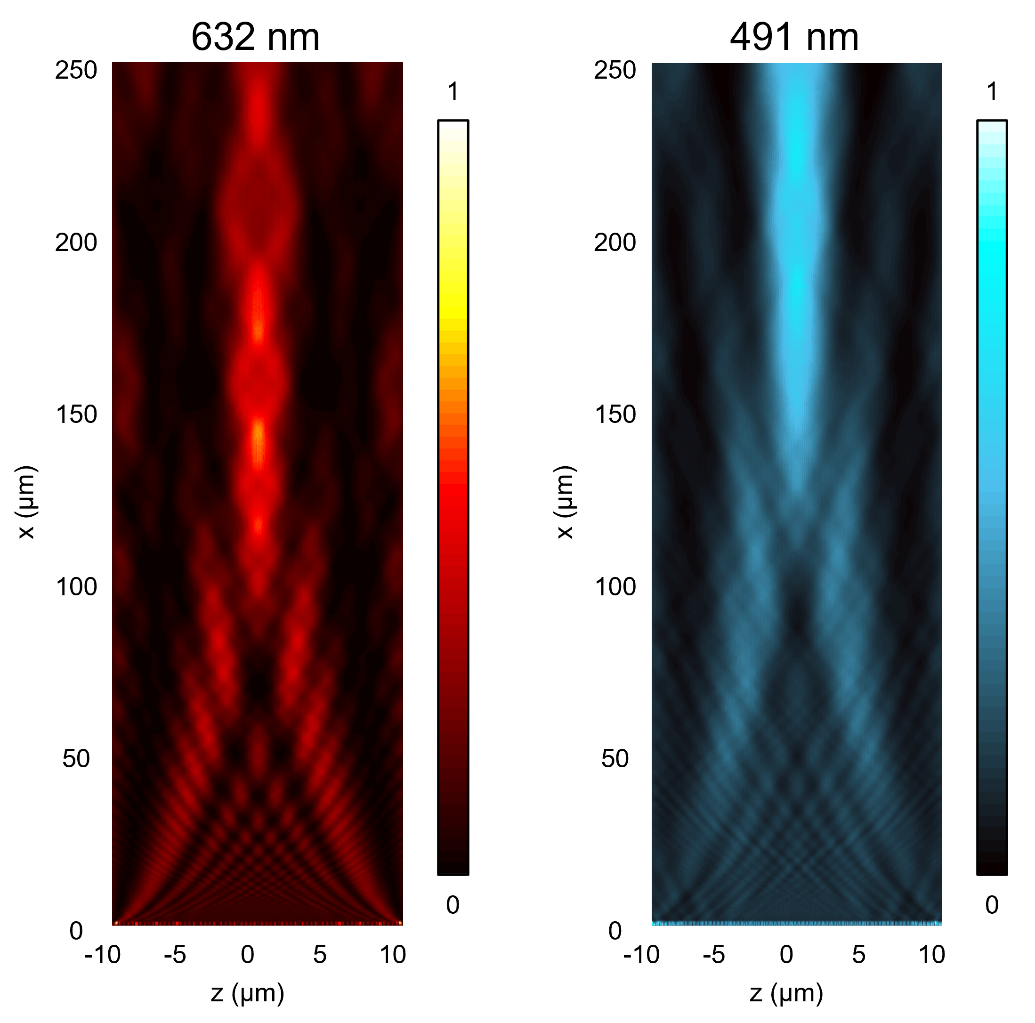


**Figure S4. The simulated beam propagation of cylindrical metalens at wavelengths of 491 and 632 nm.**

**S5. Experimental setup to measure the beam profile of the cylindrical metalens**

To measure the optical performance of fabricated cylindrical metalens, an experimental setup using a 4f system is constructed, as shown in Fig. S5. A collimated laser beam with a wavelength of 532 nm (Cobalt SambaTM 1500) is directed onto the cylindrical metalens to generate a sheet of light. A microscope system is focused on the surface of the cylindrical metalens, and the focal plane of the microscope is scanned over the lens using a linear motorized stage (NEWPORT GTS70) with step sizes of 0.02 mm and 0.001 mm. The measured images are captured using the CCD camera (Canon EOS 6D Mark Ⅱ) during the scanning process to assess the optical performance. To calculate the FOV, the equation of the theoretical Rayleigh length is shown below:

$$FOV=2\frac{\pi{w_{0}}^{2}}{\lambda}=2\frac{\pi\left( \frac{n\lambda}{\pi NA} \right)^{2}}{\lambda}$$

where $n$ is the refractive index, $\lambda$ represents the wavelength, and $NA$ is the numerical aperture of the cylindrical metalens. With the designed parameters in our setup, the theoretical Rayleigh length is 135.47 μm. The experimental measurements for both cylindrical metalenses are 153 μm and 151 μm, which is similar to the theoretical value. The measurement is achieved by calculating the beam waist range within a $\sqrt{2}$ multiple of the beam waist measured at the focal plane. The consistency between experimental and theoretical results shows the high precision of the fabricated metalens, demonstrating its suitability for various fields and applications requiring compact optical systems.


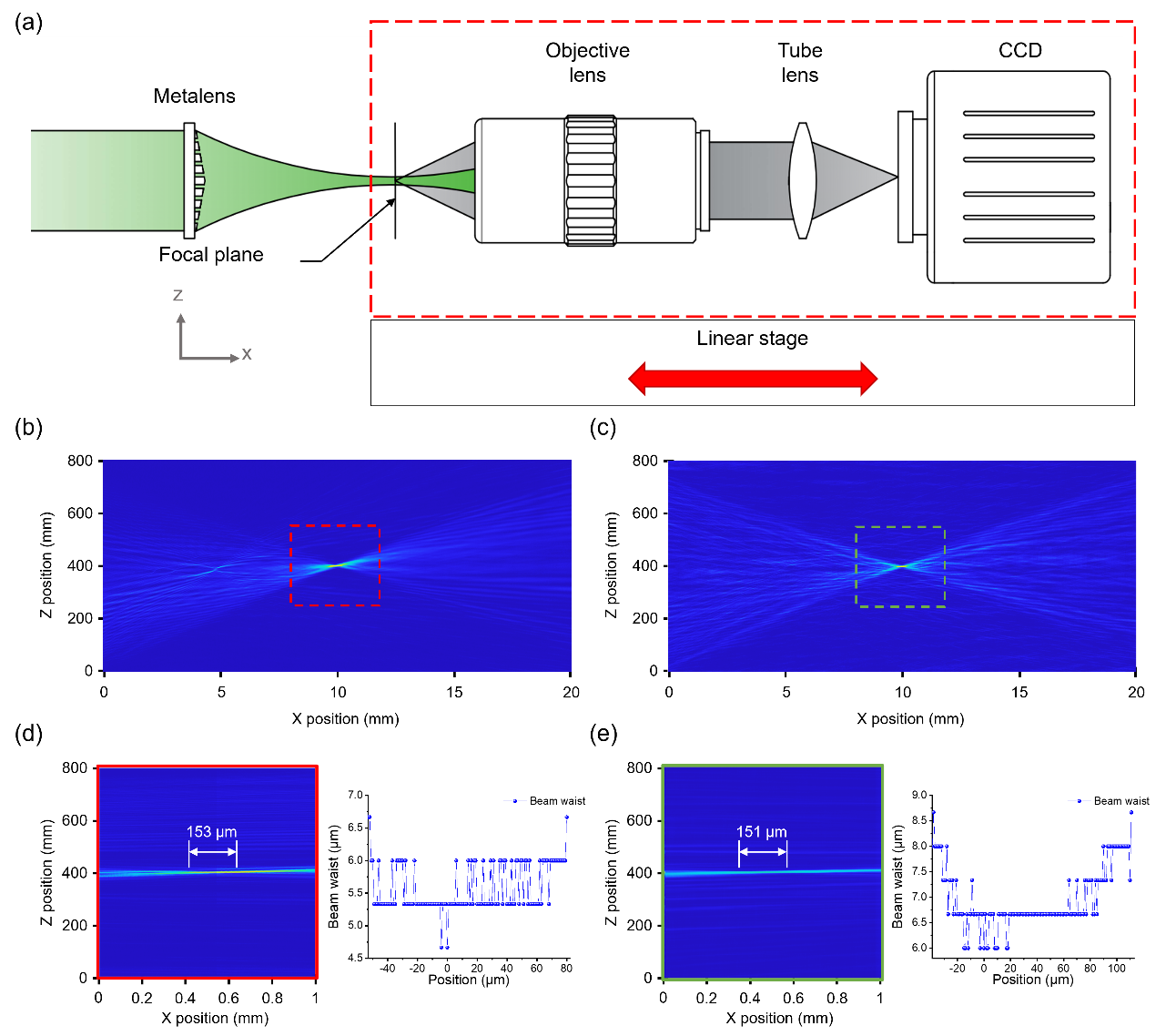


**Figure S5. Experimental setup and measured beam profiles. (a)** Schematic of the experimental setup used to measure the beam profiles of the cylindrical metalenses. **(b)** Beam propagation of the first cylindrical metalens, and **(c)** beam propagation of the second cylindrical metalens with a step size of 0.02 mm. **(d)** Beam propagation of the first cylindrical metalens, and **(e)** beam propagation of the second cylindrical metalens with a step size of 0.001 mm. The right subfigures illustrate the calculated FOV for both cylindrical metalenses.

**S6. The comparison between conventional lenses and metalens**

Figure S6 shows the comparison between the different setups, such as the objective lens plus cylindrical lens (OL+CL), cylindrical lens (CL), and cylindrical metalens (Cy meta). The cylindrical lens used in the setup has a focal length of 75 mm and a diameter of 25.4 mm, and the objective lens has an NA of 0.14. To evaluate the performance of these configurations, we use an additional 4f system and a linear translation stage with a step size of 0.02 mm, capturing 500 images in each setup. The enlarged region can be obtained by moving linear translation stage with a step size of 0.001 mm to calculate the FOV. The beam profiles observed at the focal planes of both the OL+CL and CL setups are larger than those generated by the Cy meta. The reason is that the diameter of the laser source is only 3.5 mm, which cannot cover the back aperture of the conventional lenses. An additional beam expander is necessary to address this issue, and the size of illumination is increased. However, the same specification is sufficient for the Cy meta, generating a thinner light-sheet while maintaining image quality similar to that using OL+CL and CL configurations.


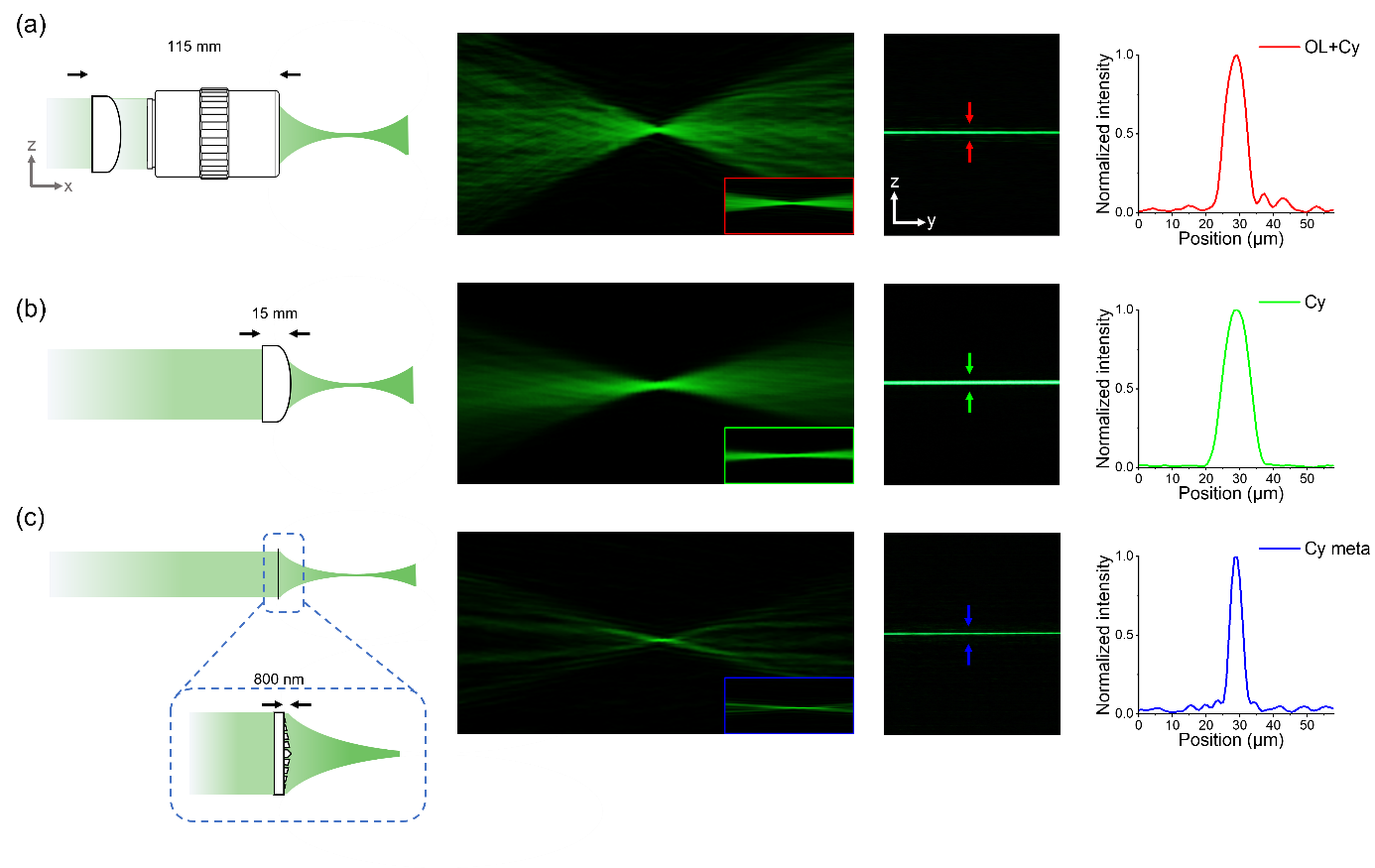


Figure S6. Comparative measurements using conventional lenses and cylindrical metalens. (a) Configuration utilizing a cylindrical lens in combination with an objective lens (OL+CL). (b) Configuration with only the cylindrical lens (CL). (c) Configuration utilizing the cylindrical metalens (Cy meta). In each subfigure, the sequence presents the experimental setup, beam propagation along the x-z with enlarged region and y-z planes at the focal plane, and the corresponding beam profile as indicated by the arrows.

**S7. The experimental setup for the proposed dual-sided illumination LSFM**

Figure S7 shows the physical structure of a dual-sided illumination for LSFM. The detection arm consists of an Olympus BX51 microscope equipped with water immersion objectives of various magnifications. The illumination arm utilizes a fiber-optic light source, which is split into bilateral illumination beams via a beam splitter and a reflector mirror. The entire illumination assembly is mounted on a z-axis piezo stage and x-y linear stages, aligning the sample setup on the corresponding linear platforms of the microscope.


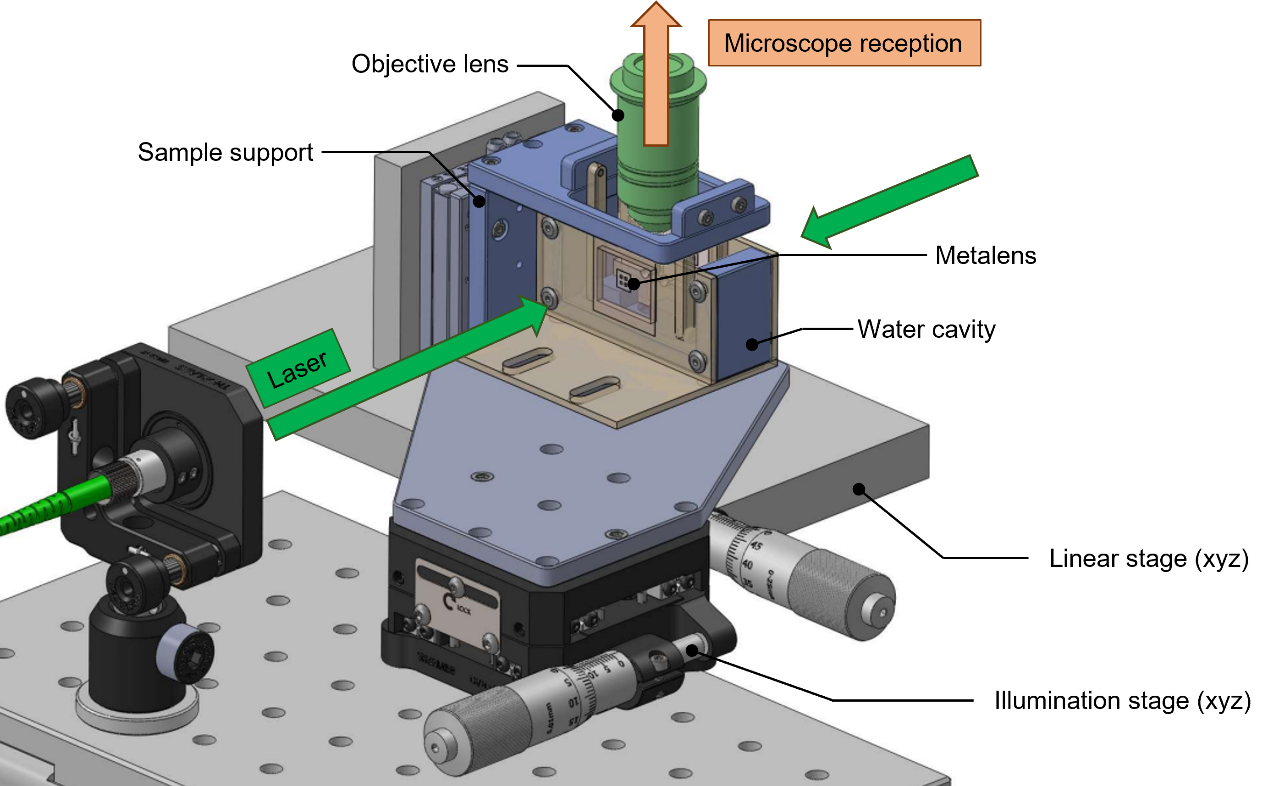


Figure S7. The experimental setup for the proposed dual-sided illumination LSFM.

References and notes

[1] J. W. Goodman, *Introduction to Fourier optics*, Roberts and Company publishers, 2005.

[2] J. Y. Yang, I. Ghimire, P. C. Wu, S. Gurung, C. Arndt, D. P. Tsai, and H. W. H. Lee, *Nanophotonics.* **2019,** 8, 443-449

[3] H. Y. Kuo, S. Vyas, C. H. Chu, M. K. Chen, X. Shi, H. Misawa, Y. J. Lu, Y. Luo, and D. P. Tsai, *Nanomaterials.* **2021,** 11, 1730

[4] C.-H. Lin, S.-H. Huang, T.-H. Lin, and P. C. Wu, *Nature communications.* **2023,** 14, 6979.

[5] P.-S. Huang, C. H. Chu, S.-H. Huang, H.-P. Su, T. Tanaka, and P. C. Wu, *Nano letters.* **2023,** 23, 10432-10440.
